# Self-sampling and HPV testing or ordinary Pap-smear in women not regularly attending screening: a randomised study

**DOI:** 10.1038/bjc.2011.236

**Published:** 2011-07-05

**Authors:** I Wikström, M Lindell, K Sanner, E Wilander

**Affiliations:** 1 Department of Women's and Children's Health, Uppsala University, S751 85 Uppsala Sweden; 2 Department of Genetics and Pathology, Uppsala University, S751 85 Uppsala, Sweden

**Keywords:** cervix, human papilloma virus, self-sampling, cytological screening

## Abstract

**Background:**

Most women with cervical cancer have not participated in Pap-smear screening. Self-sampling of vaginal fluid in combination with high-risk HPV testing may be a method to increase the attendance rate.

**Methods:**

A total of 4060 women, 39–60 years old, who had not attended the organised Pap-smear screening for 6 years or more were randomised into two equal groups. A study group was offered to self-sample vaginal fluid (Qvintip) at home and/or recommended to attend the Pap-smear screening. The collected fluid after self-sampling was examined for the presence of high-risk HPV (Hybrid Capture 2 method). Controls were only recommended to attend the Pap-smear screening. The end point was a histological identification of CIN2–3.

**Results:**

The participation rate was 39% (771 out of 2000) in the self-sampling group and 9% (188 out of 2060) in the conventional cytology (*P*<0.001). The number of histological CIN2–3 alterations detected was 0.4% (8 out of 2000) among women offered self-sampling of vaginal fluid and 0.07% (3 out of 4060) in women offered Pap-smears. The odds ratio (OR) for offering self-sampling and HPV testing instead of Pap-smear screening for detection of CIN2–3 was OR=5.42 (95% CI: 1.30–31.8).

**Conclusion:**

Offering self-sampling of vaginal fluid followed by a high-risk HPV test was considerably more effective for detection of histological CIN2–3 lesions in comparison with offering Pap-test in a midwife reception in women not regularly attending organised screening.

For documentation of the efficiency of organised Pap-smear screening for cervical cancer, nationwide audits are valuable. An investigation in Sweden showed that the majority of women (65%) with cervical cancer had not attended the organised Pap-smear screening, and around 25% developed cancer despite a regular participation due to the occurrence ‘false-negative’ smears (Andrae *et al*, 2008). A number of ‘false-negative’ smears indicates a low sensitivity of Pap-smear screening. A recent study, using primary screening with a high-risk HPV test for identification of histological CIN2–3 lesions, showed that the sensitivity of a single Pap-smear to detect CIN2–3 alterations is around 50%, also indicating a low sensitivity of cytological screening (Ronco *et al*, 2010).

To increase the participation rate and decrease the incidence of cervical cancers in the County of Uppsala, women not attending organised Pap-smear screening were, since the year 2006, offered self-sampling of vaginal fluid at home in combination with a high-risk HPV test (Stenvall *et al*, 2006; Sanner *et al*, 2009). Initially, during 2006–2007, a pilot study comprising 600 women not attending Pap-smear screening was performed (Stenvall *et al*, 2006). These women were not included in the present investigation. The HPV-positive women are recommended a follow-up in a gynaecological surgery (colposcopy clinic) or a midwife reception (family planning clinic). Preliminary results indicate that the self-sampling method is an attractive alternative for women who choose not to participate in the organised screening. Around 40% of women not regularly attending a midwife reception for smear sampling accept the home sampling method and most women attending a midwife reception for smear collection would prefer to collect vaginal fluid at home, if they were offered that possibility (Wikström *et al*, 2007a). In addition, the risk for obtaining ‘false-negative’ results is minimal (Ronco *et al*, 2010).

In this study, women not regularly attending in the organised Pap-smear screening programme were randomised into two groups of equal size. One group was offered ordinary Pap-smear screening and the other group the possibility of self-sampling of vaginal fluid at home as an alternative to Pap-smear screening. The main outcome measures of the study were the attendance rate and the identification of histological CIN2–3 lesions in both groups.

## Materials and methods

A total of 4060 women, 39–60 years old, who had not participated in the organised Pap-smear screening for 6 years or more were collected from the local data base at the Department of Pathology and Cytology, Uppsala University Hospital, Sweden in January 2007.

The women were randomly divided into a study group of 2000 women and a control group of 2060 women. In the study group, all women were offered self-sampling at home with a self-sampling device (Qvintip, Aprovix AB, Uppsala, Sweden) as an adjunct to organised Pap-smear screening. In the control group, women were only advised to participate in Pap-smear screening. In the organised Pap-smear screening, women 25–60 years old are invited for smear sampling every third year. Women, who choose not to attend receives additional invitation letters once a year.

All 2000 women in the study group were sent an information letter by post, and after a few days, they received the self-sampling device, instructions how to perform the sampling of vaginal fluid and to send the collected material to our laboratory in the enclosed, prepaid return letter. The procedure was free of charge and the women were also reminded in a second invitation. At the laboratory, the samples were used for high-risk HPV testing with the Hybrid Capture 2 (hc2) method (Qiagen AB, Solna, Sweden). The HPV test identifies 13 high-risk HPV types (16, 18, 31, 33, 35, 39, 45, 51, 52, 56, 58, 59 and 68). The hc2 method can detect HPV DNA concentrations over 1 pg ml^−1^, which is proportional to the light emission of the positive control and corresponds to 5000 HPV genomes per specimen in the well. The results of the HPV test were mailed to all women participating in the study. All information on women in the study group was collected in the database of the department, together with records on the Pap-smear screening and histopathological examinations.

The women who were high-risk HPV positive were recommended a follow-up examination at a midwife reception or a gynaecological surgery. In women examined by a gynaecologist, a biopsy from the cervix was obtained, whereas in women attending a midwife reception a repeated cytology, often in combination with a cervical sample for HPV analysis, was taken. All women offered self-sampling at home were also offered to participate in the organised Pap-smear screening.

The women in the control group were invited to a midwife reception for collection of cervical smear, within the framework of the organised screening programme. Women with ASCUS or CIN1 alterations observed in the screening were called for re-examination at the midwife reception and women with CIN2–3 cell changes were admitted to a gynaecological reception for colposcopy and cervical biopsy. The women paid 100 SEK (around 10 EUR) only for the first Pap-smear collection.

At the end of December 2007, all women who had performed self-sampling of vaginal smear at home in combination with a high-risk HPV test, and all women in the study and control groups participating in the Pap-smear screening were identified. Women who were HPV positive or showed abnormal cytology (ASCUS–CIN3) were followed until December 2009. The end point of the study was a histopathological CIN2–3 lesions observed in cervical biopsies or after cervical cone resection.

## Results

In the study group, 679 out of 2000 women (34%) accepted to perform self-sampling of vaginal fluid at home and send the collected material to our laboratory for high-risk HPV analysis, whereas 100 out of 2000 women (5%) preferred to attend a midwife reception for Pap-smear sampling. In total, 779 out of 2000 women (39%) in the study group participated in the screening. Among the controls, 188 out of 2060 women (9%) attended the Pap-smear screening programme. The difference in attendance rate between the two groups was strongly significant (*P*<0.001; Table 1).

A high-risk HPV-positive reaction was recorded in 41 out of 679 women (6.0%) performing self-sampling. The prevalence of HPV infection decreased with age, it was 7.5% (23 out of 305) in women 39–49 years old and 4.8% (18 out of 374) in women aged 50–60 years.

Of HPV-positive women, 24 out of 41 (59%) visited a gynaecological surgery, directly or via a midwife reception, for further examination including colposcopy and biopsy within 1–7 months after the collection of vaginal fluid. A total of 16 out of 41 (39%) only visited a midwife reception. The compliance of the HPV-positive women was 98% (40 out of 41). One woman had moved out of the county and was not possible to reach.

The biopsies showed normal histology in 12 cases, CIN1 in 4 cases and CIN2–3 in 8 cases (Table 2). Women with CIN2–3 lesions were treated with cervical cone resection, whereas women with CIN1 and 4 women with normal histology and a persistent HPV-positive reaction were offered a continuous follow-up. Of the 16 women visiting only a midwife reception, 1 was HPV positive and 1 showed ASCUS. They were followed up, and in no case, a histological CIN2–3 was recorded.

A total of 100 women in the study group and 188 of the controls participated in the Pap-smear screening. Of these women, one cytological slide was not representative, one showed ASCUS, one CIN1 and three CIN2–3. The women with ASCUS were HPV negative, the women with CIN1 had a normal biopsy and the women with CIN2–3 were operated with cone resection, which histologically verified the CIN2–3 lesions.

All 4060 women in the investigation were offered collection of Pap-smear at a midwife reception, and 288 chose to participate. Of the participating women, three were identified with histological CIN2–3 on the cervix. A total of 2000 women were offered self-sampling of vaginal fluid at home in combination with HPV testing, and of 679 participating women, 8 had histological CIN2–3 lesions on the cervix. The odds ratio (OR) for identification of histological CIN2–3 lesions with the self-sampling at home method in comparison with Pap-smear screening was OR=5.42 (95% CI: 1.3–31.8; Table 3).

## Discussion

As mentioned in the introduction, non-attendance is the major problem in countries with an organised Pap-smear screening for cervical cancer (Bos *et al*, 2006; Andrae *et al*, 2008; Lindqvist *et al*, 2008). Although this fact has been known for many years, no simple method to increase the coverage to the cytological screening is available (Eaker *et al*, 2004; Oscarsson *et al*, 2008). However, combinations of different invitations are reported to increase the number of detected precursor lesions (Eaker *et al*, 2004).

The method of self-sampling of vaginal fluid at home seems to be an attractive alternative for the non-participating women. It is less time consuming and it offers a possibility to avoid the gynaecological chair position. The HPV test is also about twice as sensitive as a Pap test to identify histological CIN2–3 lesions. Several studies have shown that 30–50% of women not attending midwife receptions for Pap-smear screening accept the self-sampling method (Stenvall *et al*, 2006; Sanner *et al*, 2009; Gök *et al*, 2010). In this study, 87% of the attending women preferred self-sampling when they had the possibility to choose between self-sampling and Pap-smear screening. The fact that the self-sampling alternative was free of charge has probably contributed to the high attendance rate in this group. However, among women visiting a gynaecological surgery, 90% explained that they would prefer self-sampling at home instead of Pap-smear collection at a midwife reception provided that this possibility was available (Wikström *et al*, 2007a).

Although there is a general interest in increasing the coverage of the organised Pap-smear screening, there are also some objections against the use of HPV tests in organised screening for cervical cancer. One main reason is that, despite the high sensitivity, the HPV test is considered to have a too low specificity (Wright *et al*, 2004; Meijer *et al*, 2009). A number of women will be identified with high-risk HPV infection but without any cytological alterations, and no method is available for treatment of the HPV infection.

However, it must be kept in mind that the prevalence of high-risk HPV infections decreases with age and in post-menopausal women HPV infections are almost as uncommon as cell alterations (ASCUS–CIN3) in the Pap-smear screening. Consequently, in women 50 years and older HPV tests are almost as specific as Pap-smear screening (Wikström *et al*, 2007b; Sanner *et al*, 2009). Furthermore, the sensitivity of conventional cytology decreases markedly in post-menopausal women and most women with CIN2–3 lesions on the cervix display normal cytology (Gustafsson *et al*, 1995; Gyllensten *et al*, 2010). For that reason, self-sampling of vaginal fluid at home and HPV testing seems to be a reliable method to increase the attendance rate and also the sensitivity of the screening in countries with an organised Pap-smear screening programme. It is also indicated that self-sampling and HPV testing may be an attractive method for screening of menopausal women, an age category in which only around 20% of the maximal effect of Pap-smear screening remains and in which most women with cell alterations (ASCUS–CIN3) are high-risk HPV negative (Gyllensten *et al*, 2010).

A power analysis was performed. The number of participating women was however limited by the financial support for the study. The study population is too small to document differences in sensitivity between HPV and Pap-smear screening but it shows that self-sampling at home is an alternative to ordinary repeated calling of Pap-smear collection to non-responders.

## Figures and Tables

**Table 1:** Recruitment of 39- to 60-year-old women not attending organised cytological screening for more than 6 years by invitation for self-sampling of vaginal smear at home and/or Pap-smear screening (study group, 2000 cases) and offering only Pap-smear screening (control group, 2060 cases)

**Categories**	**Study group**	**Controls**
Total number of women	2000	2060
Self-sampling of vaginal fluid	679	0
Pap-smear screening	100	188
Not attending	1221	1872
Total number of participating women	779/2000 (39%)	188/2060 (9.1%)^*^

^*^P<0.001.

**Table 2:** Light microscopic morphology in 24 cervical biopsies of high-risk human papilloma virus-positive women

**Light microscopy**	**Number of cases**
Normal	12/24 (50 %)
CIN1	4/24 (17%)
CIN2–3	8/24 (33%)^a^

a One case with only cytological findings of CIN3.

**Table 3:** Number of women with histological CIN2–3 lesions among women offered self-sampling of vaginal smear at home in combination with high-risk human papilloma virus testing and in women offered organised Pap-smear screening

**Categories**	**Self-sampling of vaginal fluid**	**Pap-smear**
Total number of women	2000	4060
Number of participating women	679/2000 (34%)	288/4060 (7.1%)
Number of women with CIN2–3	8/679 (1.2%)^a^	3/288 (1.0%)
Number of CIN2–3/total number of women	8/2000 (0.4%)^b^	3/4060 (0.07%)^b^

a One case with only cytological findings of CIN3.

b Odds ratio=5.42 (95% confidence interval: 1.30–31.8).
